# Detecting short-term change and variation in health-related quality of life: within- and between-person factor structure of the SF-36 health survey

**DOI:** 10.1186/s12955-015-0395-1

**Published:** 2015-12-21

**Authors:** Amanda Kelly, Jonathan Rush, Eric Shafonsky, Allen Hayashi, Kristine Votova, Christine Hall, Andrea M. Piccinin, Jens Weber, Philippe Rast, Scott M. Hofer

**Affiliations:** Department of Psychology, University of Victoria, 3800 Finnerty Road, Victoria, BC V8P 5C2 Canada; Family Physician, 2020 Richmond Road, Victoria, BC V8R 6R5 Canada; Pediatric and General Surgery, Island Health, 1952 Bay Street, Victoria, BC V8R 1J8 Canada; Department of Research, Island Health, Victoria, BC Canada; Emergency Department, Island Health, Victoria, BC Canada; Department of Computer Science, University of Victoria, Victoria, BC Canada

**Keywords:** Patient-reported outcomes, Health-related quality of life, SF-36 health survey, Multilevel confirmatory factor analysis

## Abstract

**Background:**

A major goal of much aging-related research and geriatric medicine is to identify early changes in health and functioning before serious limitations develop. To this end, regular collection of patient-reported outcome measure (PROMs) in a clinical setting may be useful to identify and monitor these changes. However, existing PROMs were not designed for repeated administration and are more commonly used as one-time screening tools; as such, their ability to detect variation and measurement properties when administered repeatedly remain unknown. In this study we evaluated the potential of the RAND SF-36 Health Survey as a repeated-use PROM by examining its measurement properties when modified for administration over multiple occasions.

**Methods:**

To distinguish between-person (i.e., average) from within-person (i.e., occasion) levels, the SF-36 Health Survey was completed by a sample of older adults (N = 122, *M*_*age*_ = 66.28 years) daily for seven consecutive days. Multilevel confirmatory factor analysis (CFA) was employed to investigate the factor structure at both levels for two- and eight-factor solutions.

**Results:**

Multilevel CFA models revealed that the correlated eight-factor solution provided better model fit than the two-factor solution at both the between-person and within-person levels. Overall model fit for the SF-36 Health Survey administered daily was not substantially different from standard survey administration, though both were below optimal levels as reported in the literature. However, individual subscales did demonstrate good reliability.

**Conclusions:**

Many of the subscales of the modified SF-36 for repeated daily assessment were found to be sufficiently reliable for use in repeated measurement designs incorporating PROMs, though the overall scale may not be optimal. We encourage future work to investigate the utility of the subscales in specific contexts, as well as the measurement properties of other existing PROMs when administered in a repeated measures design. The development and integration of new measures for this purpose may ultimately be necessary.

## Background

A chief goal of aging-related research and geriatric medicine is to enhance quality of life with the identification of early changes in health and functioning that may herald more serious problems in the future, and to intervene before serious limitations develop. A potential avenue to monitor these changes is through regular collection of patient-reported outcome measures (PROMs), self-reports related to symptoms (e.g., type, frequency, severity, duration), functioning (e.g., health limitations, activities of daily living), perceptions (e.g., satisfaction with treatment) and overall well-being. The early cognitive, behavioral and physical changes that characterize advancing age are difficult to detect and may vary from day to day within individuals [1]. Consequently, opportunities to provide intervention efforts at clinically relevant times are diminished which may reduce possibilities for prevention and advanced care planning. The assessment of change and variation over time using PROMs is promising but an under-examined area of the literature.

Both patient self-report and direct measurement of functioning can be collected regularly, in a clinic or home setting, to monitor a patient’s health and change in functioning. Based on the premise that the most robust clinical approaches for detecting change or the effects of clinical interventions require repeated measurements, regular collection of PROMs is a means to establish stable patient baselines against which fluctuations and systematic changes are identifiable and used to trigger clinical interventions. Frequent assessment provides less biased and more representative sampling of patient symptoms, functioning and quality of life indicators than single assessments which are susceptible to recall bias and other errors [2]. In addition, measures on a single point in time evaluate scores against norm reference standards and their clinical sensitivity/specificity is inherently limited by the heterogeneity of the populations within which they are employed. However, very few research studies have investigated the utility and impacts of repeated PROM administration as a means for enabling the individual to establish their own baseline or reference standard. Repeated assessment of PROMs permits the detection of individual change, such as in response to treatment, with a high degree of sensitivity and with the interpretation of change relative to their own prior level rather than the more typical normative between-person interpretation.

Despite the measurement limitations inherent in the norm-referenced paradigm, one-time and short-term use of PROMs has demonstrated improvements in patient satisfaction, well-being and autonomy, patient-physician communication and the detection of mental health diagnoses [3–6]. In addition, patient reports have been found to contribute unique predictive power to models of mortality [7] and in some cases are more informative than physician ratings of patient status [8–10], which may reflect the broader impacts of symptoms on everyday life and overall well-being captured by self-reports [7]. As such, regular utilization of PROM data within the health care system has foreseeable benefits for both clinical practice and research, and may provide an important complement to clinician-derived health data. With recent wider adoption of electronic health records and renewed patient-centred focus, Wu and colleagues [11] have argued that the integration of PROMs in health care is now more feasible than ever before. Despite these calls for action [12], PROMs are not typically part of routine clinical care appointments or standard prognostic assessments [13, 14], with few exceptions (e.g., [15]). This may result in a loss of relevant individualized patient information which may be useful to complement health records, guide medical decision-making and patient management, and inform research efforts [16, 17].

Clinical use of PROMs may prove useful to improve patient health behaviours, outcomes and patient management, though evidence is mixed (e.g., [18, 19]). In large part, this lack of consensus is limited by a general lack of repeated measure studies to drive evidence-based medicine; related to this, the psychometric properties of PROMs when administered over multiple occasions remains largely unknown. Given that the majority of these measures were designed for one-time use to compare between-person (i.e., average) differences, their suitability to monitor fluctuations and systematic changes should not be taken for granted [20]. However, while the development of new measures is a time-consuming process, the adaptation and evaluation of an already-available measure may be a more practicable approach to achieving the goal of regular in-clinic patient assessment. Examples include the NIH-funded Patient-Reported Outcomes Measurement Information System [21], the Functional Assessment of Chronic Illness Therapy questionnaires [22], the Health Assessment Questionnaire [23], the RAND 36-Item Short Form Health Survey (SF-36; [24]) and disease-specific scales such as the Diabetes Health Profile [25]. The SF-36 is brief, disease-generic and readily available free of charge (version 1.0); despite critiques of its measurement properties [26–30] its ubiquity and its conceptualization of health as a well-rounded concept comprised of eight domains make it a suitable first candidate for this investigation.

Two major research questions remain to be answered, and are addressed in the present study. First, is the SF-36 sensitive enough for reliable detection of short-term variation at the within-person (i.e., occasion) level? This demonstration is an important first step in the selection of a measure to monitor long-term change. The second question concerns the psychometric properties of the SF-36 when administered over multiple occasions: when used in this new way, are its factor structure and goodness of fit indices different from standard administration? To further explore these questions, the present study applied an intensive repeated measure design to disaggregate within-person variance (i.e., daily deviations from personal levels) from between-person variance. This approach allows simultaneous but separate modeling of the daily within-person fluctuations and the between-person differences to yield both within-person and between-person factor structures. Such a continuously intensive design is likely not feasible in real-world applications of PROMs, where wider assessments repeated at regular intervals in the context of a measurement burst design would suffice to broadly capture person-level change by distinguishing short-term variations from long-term changes [31, 32]. As of yet, the within-person structure of the SF-36 remains unexamined, though this analysis is central to our understanding of its potential as a repeated-use PROM.

## Methods

### Sample

We recruited 122 older adults through advertisements placed in a local family health clinic seeking people aged 50 and older for research on health and well-being during aging. The sample for analysis had a mean age of 66.28 years (*SD* = 8.57, range: 50–88), was evenly split between the sexes (55 % female) and rated general health as good on the SF-36 general health item (*M* = 6.73, *SD* = 1.62). All participants provided informed consent to participate and ethical approval was obtained from the University of Victoria and Vancouver Island Health Authority Joint Research Ethics Sub-Committee (protocol number J2012-70).

### Measures

The RAND 36-Item Short Form Health Survey (SF-36; version 1.0) was developed at RAND Health as part of the Medical Outcomes Study. It is a brief and easily-administered measure of health-related quality of life and consists of 36 multiple-choice items assessing eight health domains: physical functioning; role limitations due to physical health; role limitations due to emotional problems; vitality; mental health; social functioning; bodily pain and general health. Summary physical component and mental component summary scores can also be computed. Scores for each domain range from 0 to 100 where 100 indicates an excellent health state and no reported symptoms. This simple linear transformation was performed to improve interpretation of small estimates.

### Procedures

Participants completed the standard SF-36 at baseline and provided up to seven responses on consecutive days to the survey modified for repeated administration. This simple modification instructed participants to respond based on the previous 24 h by adjustment of the timescale to which items referred. Exemplars are presented in Table 1. Items 33-36 under the General Health subscale were not included in the daily survey as they were not relevant to daily experience. Data from two items were lost due to technical difficulties with the electronic medical record system used for data collection. Of a possible 854 total assessments (122 patients X 7 days), 694 complete observations were obtained (81 %; *M* = 5.69) which provides sufficient statistical power for our analyses. Each session was completed via computer through a web-based patient portal survey tool and required approximately 20 min daily. Though burden can be a concern in intensive repeated measures designs such as this, over 96 % of participants reported willingness to take part in similar future studies, an indication that the time commitment was not too great.

**Table 1:** Original SF-36 exemplar items and modification for daily survey

SF-36 subscale	Original wording	Modified wording
Physical functioning	The following items are about activities you might do during a typical day. Does your health now limit you in these activities? If so, how much?	The following items are about activities you might do during a typical day. Did your health limit you in these activities during the past 24 h? If so, how much?
Bodily pain	How much bodily pain have you had during the past 4 weeks?	How much bodily pain have you had during the past 24 h?
Vitality, mental health	These questions are about how you feel and how things have been with you during the past 4 weeks. For each question, please give the one answer that comes closest to the way you have been feeling. How much of the time during the past 4 weeks . . .	These questions are about how you feel and how things have been with you during the past 24 h. For each question, please give the one answer that comes closest to the way you have been feeling. How much of the time during the past 24 h . . .

*Note. SF-36* RAND Medical Outcomes Study Short-Form Health Survey 1.0

### Analytic approach

To evaluate our first research question, the ability of the SF-36 to detect short-term variation at the within-person level, we computed intraclass correlation coefficients (ICC) for the 30 items and eight subscales of the survey. This metric provides the proportion of between-person variance to total variance. The remaining proportion of the variability (i.e., 1-ICC) gives an indication of the amount of within-person variability. Thus interpretation of the ICC can be summarized as small values (i.e., <0.50) indicating items which capture more within- (i.e., occasion) than between-person (i.e., average) variation.

To evaluate our second research question, confirmatory factor analyses (CFA) were run based on the published eight-factor (eight subscales among the 36 items) and two-factor (two summary component scores among the 36 items) SF-36 structure [33]. Single-level CFA models were run for the standard survey administered at baseline and multilevel CFAs were run for the modified survey administered daily to evaluate both the within-person and the between-person factor structure. In multilevel factor analysis, the within-person factor structure reflects common covariance among the items on each specific day, pooled across days and individuals. The between-person factor structure reflects common covariance in individual mean levels of each item aggregated across time [34].

Goodness of fit and sources of model misfit were examined with the comparative fit index (CFI), Tucker-Lewis index (TLI), root-mean-square error of approximation (RMSEA) and standardized root-mean-square residual (SRMR). Ideal values range from .90 (acceptable) to greater than .95 (good) for CFI and TLI; and from .08 (plausible/acceptable) to less than .05 (good) for RMSEA and SRMR [35]. While the CFI, TLI and RMSEA are indicators of overall fit, the SRMR provides separate fit indices for both the within- and between-person levels. All models were estimated using Mplus Version 7 [36] with maximum likelihood for robust estimates (MLR).

## Results

### Detection of within-person variability by the daily SF-36

**Fig. 1:**
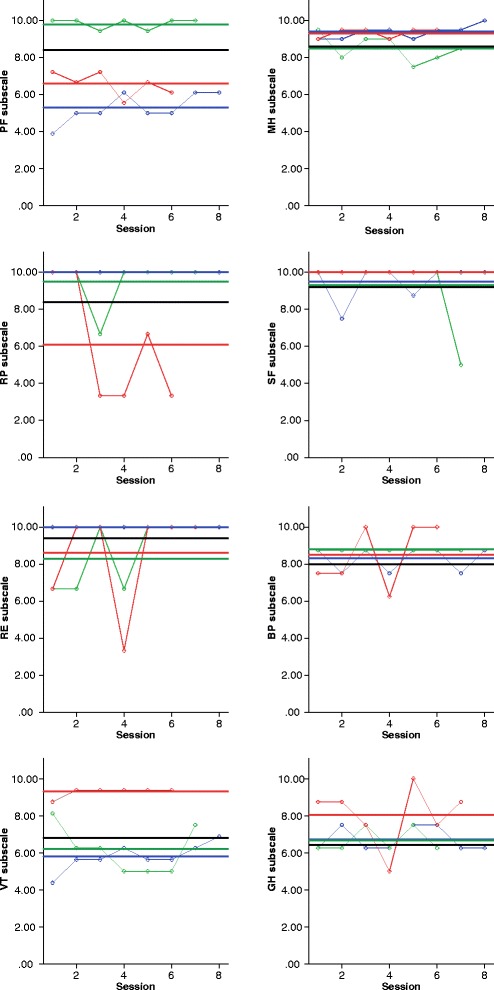
Panel plot illustrating between- and within-person variability across subscales on the daily SF-36. Thin lines indicate raw scores across sessions for three randomly selected participants. Thick lines indicate person-mean and sample-mean (*black*) scores. *Note*. PF = physical functioning; RP = role physical; RE = role emotional; VT = vitality; MH = mental health; SF = social functioning; BP = bodily pain; GH = general health

Means, standard deviations and ICC values for the 30 items and eight subscales of the daily SF-36 are presented in Table 2. At the subscale level, we observed a wide range of ICC values, an indication that some subscales captured more daily fluctuations than others. The Emotional Role Limitations subscale with an ICC of .38 captured the largest proportion (62 %) of within-person variability; that is, response patterns to this subscale were more closely aligned with occasion-specific fluctuations than with stable differences between individuals. On the other hand, the physical functioning subscale with an ICC of .89 captured only 11 % of within-person variability. The majority of the variance in responses to this subscale was due to between-person differences. As with the subscales, item-level ICC values ranged from .09 for *yesterday* (General Health; 91 % within-person variability) to .84 for *walk mile* (Physical Functioning; 16 % within-person variability). Items within the same subscale generally exhibited similar proportions of within-person variation with only a few exceptions (see the Physical Functioning and General Health subscales). Figure 1 provides an illustration of the extent of dynamic, within-person variation from day to day on each SF-36 subscale (variation around the person-mean), as well as the degree of stable, between-person differences (variation around the sample mean).

**Table 2:** Means, standard deviations, intraclass correlation coefficients and reliability estimates (ω) for the baseline (standard) and daily administrations of the SF-36

	Baseline SF-36			Daily SF-36				
Variable	Mean	SD	Omega (BP)	Mean	SD	ICC	Omega (BP)	Omega (WP)
Physical functioning	81.4	21.7	.92	84.3	22.8	.89	.96	.70
Vigorous	42.8	38.4		58.0	41.0	.81		
Moderate	82.0	31.9		83.4	31.6	.83		
Climb several	75.4	35.7		80.4	34.5	.82		
Climb one	92.0	22.0		92.8	22.3	.68		
Bending	75.8	31.1		81.0	31.2	.81		
Walk mile	81.8	33.4		80.5	35.3	.84		
Walk several blocks	88.1	27.7		87.8	28.4	.81		
Walk one block	96.1	15.0		95.1	18.9	.73		
Bathing	98.0	11.8		97.7	12.6	.52		
Role physical	78.6	35.7	.84	84.0	32.1	.62	.96	.68
Cut down	58.4	35.3		87.6	32.9	.52		
Accomplished	74.6	43.6		82	38.5	.51		
Limited	77.0	42.1		82.5	38.0	.62		
Role emotional	84.7	29.7	.81	93.7	20.1	.38	.90	.73
Cut down	88.7	31.7		95.0	21.8	.32		
Accomplished	77.9	41.5		89.6	30.6	.34		
Not careful	87.5	33.1		96.6	18.1	.36		
Vitality	65.1	17.3	.82	68.1	21.7	.73	.95	.74
Pep	60.2	21.4		59.7	25.9	.70		
Energy	59.5	22.9		61.1	26.0	.65		
Worn-out	76.8	21.7		81.7	23.7	.66		
Tired	64.2	20.0		69.9	23.6	.58		
Mental health	80.0	13.4	.76	86.2	12.6	.56	.90	.60
Nervous	84.1	21.6		92.9	17.1	.44		
Dumps	93.1	15.2		96.7	13.7	.31		
Calm	63.9	18.5		70.7	22.4	.52		
Blue	85.1	18.7		93.8	15.6	.35		
Happy	73.4	16.0		76.7	19.7	.50		
Social functioning	87.9	19.2	^a^	91.6	18.7	.57	^a^	^a^
Extent	88.9	18.9		92.4	18.4	.52		
Time	86.9	21.4		90.9	22.4	.48		
Bodily pain	73.5	23.1	^a^	80.4	22.7	.81	^a^	^a^
Magnitude	84.7	21.0		72.5	27.4	.80		
Interfere	61.7	28.5		88.3	20.9	.74		
General health	67.3	16.2	.81	64.2	13.9	.41	^a^	^a^
In general/Today	70.2	21.4		73.3	21.1	.73		
Last year/Yesterday	52.5	17.6		55.0	15.9	.09		
Easier	84.6	22.6		-	-	-		
Healthy	74.0	27.2		-	-	-		
Worse	51.8	28.2		-	-	-		
Excellent	71.5	26.3		-	-	-		

*Note. ICC* intraclass correlation coefficient, *WP* within-person, *BP* between-person

^a^Reliability could not be computed due to insufficient number of items for the subscale

Within- and between-person reliability estimates were computed for each subscale from the application of the multilevel omega (*ω*) to both levels of the multilevel CFA models [37]. Reliability estimates could not be computed for three subscales (i.e., Social Functioning, Bodily Pain and General Health) due to an insufficient number of items per subscale. Within-person reliability estimates ranged from .60 (Mental Health) to .74 (Vitality). Between-person reliability ranged from .90 (Mental Health) to .96 (Physical Functioning) and was consistently higher for the daily SF-36 than for the standard SF-36, which ranged from .76 (Mental Health) to .92 (Physical Functioning).

### Within- and between-person factor structure of the standard and daily SF-36

Factor loadings and goodness of fit indices for the correlated eight-factor and two-factor models are presented in Tables 3 and 4, respectively.

**Table 3:** Standardized factor loadings and goodness of fit indices from multilevel confirmatory factor analyses of the baseline (standard) and daily administrations of the SF-36 (correlated 8-factor model)

	Baseline SF-36		Daily SF-36	
Variable	WP	BP	WP	BP
Physical functioning	**-**	**-**	**-**	**-**
Vigorous	-	.58	*.10*	.71
Moderate	-	.77	.42	.91
Climb several	-	.84	.37	.93
Climb one	-	.82	.80	.90
Bending	-	.70	.43	.75
Walk mile	-	.88	*.29*	.96
Walk several blocks	-	.86	.69	.95
Walk one block	-	.70	.80	.80
Bathing	**-**	.49	.68	.45
Role physical	-	**-**	**-**	**-**
Cut down	-	.78	.70	.88
Accomplished	-	.72	.68	.91
Limited	-	.89	.54	.99
Role emotional	-	**-**	**-**	**-**
Cut down	-	.81	.85	.95
Accomplished	-	.82	.65	.87
Not careful	-	.62	.61	.81
Vitality	**-**	**-**	**-**	**-**
Pep	-	.95	.74	.97
Energy	-	.82	.74	1.00
Worn-out	-	.53	.57	.78
Tired	-	.56	.51	.88
Mental health	-	**-**	**-**	**-**
Nervous	-	.49	.69	.59
Dumps	-	.77	.68	*.44*
Calm	-	.62	*.25*	.89
Blue	**-**	.66	.66	.75
Happy	-	.70	*.28*	.89
Social functioning	-	**-**	**-**	**-**
Extent	-	.92	.76	1.02
Time	-	.88	.50	.93
Bodily pain	-	**-**	**-**	**-**
Magnitude	-	.92	.50	.86
Interfere	-	.78	.82	1.02
General health	-	**-**	**-**	**-**
In general/Today	**-**	.87	.63	1.06
Last year/Yesterday	-	*.09*	.50	*-.22*
Easier	-	.51	-	-
Healthy	-	.74	-	-
Worse	-	.52	-	-
Excellent	-	.90	-	-
Goodness of fit				
CFI^a^	.83		.73	
TLI^a^	.81		.69	
RMSEA^a^	.08		.06	
SRMR	-	.08	.08	.11

*Note.* Overall fit index. Non-significant factor loadings are italicized

*WP* within-person, *BP* between-person, *CFI* comparative fit index, *TLI* Tucker-Lewis index, *RMSEA* root-mean-square-error of approximation, *SRMR* standardized root-mean-square residual

^a^Indicators of overall fit

**Table 4:** Standardized factor loadings and goodness of fit indices from multilevel confirmatory factor analyses of the baseline (standard) and daily administrations of the SF-36 (correlated 2-factor model)

	Subscale	Baseline SF-36		Daily SF-36	
Variable		WP	BP	WP	BP
Physical summary component		**-**	**-**	**-**	**-**
Vigorous	Physical Functioning	-	.62	*.25*	.73
Moderate	Physical Functioning	-	.79	.50	.90
Climb several	Physical Functioning	-	.83	.46	.91
Climb one	Physical Functioning	-	.75	.69	.84
Bending	Physical Functioning	-	.68	.42	.77
Walk mile	Physical Functioning	-	.86	.42	.94
Walk several blocks	Physical Functioning	-	.79	.64	.91
Walk one block	Physical Functioning	-	.64	.66	.73
Bathing	Physical Functioning	**-**	.49	.58	.48
Cut down	Physical Role Limitations	-	.54	*.37*	.63
Accomplished	Physical Role Limitations	-	.60	*.31*	.74
Limited	Physical Role Limitations	-	.67	*.32*	.75
Magnitude	Bodily Pain	-	.80	*.38*	.75
Interfere	Bodily Pain	**-**	.67	.58	.83
In general/Today	General Health	-	.58	*.41*	.64
Last year/Yesterday	General Health	-	.25	.30	*-.25*
Easier	General Health	-	.34	-	-
Healthy	General Health	-	.45	-	-
Worse	General Health	-	.40	-	-
Excellent	General Health	-	.63	-	-
Mental summary component					
Cut down	Emotional Role Limitations	**-**	.58	.63	*.68*
Accomplished	Emotional Role Limitations	-	.59	.58	*.63*
Not careful	Emotional Role Limitations	-	.55	.51	*.60*
Pep	Vitality	-	.73	.62	.87
Energy	Vitality	-	.59	.60	.91
Worn-out	Vitality	-	.57	.50	.88
Tired	Vitality	-	.54	.42	.91
Nervous	Mental Health	-	.49	.34	*.39*
Dumps	Mental Health	-	.69	.32	*.23*
Calm	Mental Health	-	.61	.46	.65
Blue	Mental Health	**-**	.60	.46	*.52*
Happy	Mental Health	-	.61	.48	.67
Extent	Social Functioning	-	.81	.61	.80
Time	Social Functioning	-	.82	.40	.74
Goodness of fit					
CFI^a^		.65		.43	
TLI^a^		.62		.40	
RMSEA^a^		.12		.08	
SRMR		-	.11	.10	.17

*Note.* Overall fit index. Non-significant factor loadings are italicized

*WP* within-person, *BP* between-person, *CFI* comparative fit index, *TLI* Tucker-Lewis index, *RMSEA* root-mean-square-error of approximation, *SRMR* standardized root-mean-square residual

^a^Indicators of overall fit

The eight-factor model fit to the standard SF-36 administered at baseline was not optimal as per Hu and Bentler’s criteria [35]. All items loaded onto their respective subscale factor with the exception of one item under General Health (*last year*). Significant, moderate-to-high correlations were observed between all eight factors (range *r =* .41 to .80, *p*s < .001). Model fit was not substantially different for the modified SF-36 administered daily; all items loaded significantly onto their respective subscale factor with the exception of one item at the between-person level (*yesterday* under General Health) and three items at the within-person level (*vigorous* under Physical Functioning; *calm* and *happy* under Mental Health). All factors were significantly correlated at the between-person level (range *r* = .34 to .84, *p*s < .01). Within-person factor correlations were smaller (range *r* = .29 to .85, *p*s < .01) and not found between the Mental Health factor and others, or between the Physical Functioning and Physical or Emotional Role Limitations factors (see Table 5).

**Table 5:** Between-person and within-person correlation coefficients between subscales of the eight-factor solution for the baseline (standard) and daily administrations of the SF-36

Subscale	Baseline survey							
	Physical functioning	Physical role limitations	Emotional role limitations	Vitality	Mental health	Social functioning	Bodily pain	General health
Physical functioning	-							
Physical role limitations	.64	-						
Emotional role limitations	.45	.56	-					
Vitality	.46	.53	.47	-				
Mental health	.43	.48	.74	.63	-			
Social functioning	.50	.71	.60	.67	.78	-		
Bodily pain	.80	.65	.50	.53	.53	.57	-	
General health	.54	.49	.41	.73	.59	.64	.61	-
	Daily Survey							
Physical functioning	-	*.21*	*.26*	.29	*.09*	.53	.54	.39
Physical role limitations	.69	-	.34	.42	*.11*	.49	.53	.37
Emotional role limitations	.58	.76	-	.42	.42	.58	.34	.45
Vitality	.47	.62	.51	-	*.37*	.55	.44	.85
Mental health	.34	.44	.68	.69	-	*.25*	*.04*	*.31*
Social functioning	.56	.78	.79	.64	.61	-	.49	.70
Bodily pain	.75	.84	.75	.55	.55	.75	-	.44
General health	.55	.60	.58	.69	.69	.61	.59	-

*Note*. Between-person correlations are below the diagonal. Within-person correlations are above the diagonal. Non-significant correlations are italicized

For both versions of the SF-36, overall model fit as assessed by the CFI, TLI and RMSEA was better in the eight-factor model than in the two-factor model representing physical and mental summary components. All items in the standard SF-36 loaded onto their respective summary component, but the within-person factor loadings of several physical summary scale items on the daily SF-36 were non-significant. This includes all of the Physical Role Limitations items and one each under Physical Functioning, Bodily Pain and General Health. At the between-person level in the daily SF-36, only *yesterday* under General Health did not load onto the physical summary while the Emotional Role Limitations items and two Mental Health items did not load onto the mental summary factor. Both summary factors were significantly correlated to a moderate-high degree in both the standard (*r* = .68, *p* < .001) and daily (*r*_*within*_ = .61, *p* < .05; *r*_*between*_ = .63, *p* < .05) SF-36 models. As per the hypothetical factor structure originally proposed by Ware, Kosinski and Keller [38], orthogonal models were evaluated for both solutions. Model fit across all indices was poor (results not shown here).

## Discussion

In recent years, the integration of PROMs into clinical practice to improve health outcomes and the patient experience [3–6] has been increasingly recognized as a worthwhile pursuit feasible through the use of electronic medical record systems [11–13]. Regular use may facilitate the identification of early changes that may herald more serious health problems in the future, and may provide opportunities for clinical intervention. The first step to achieving this goal and evaluating its impact is to investigate which measures are best able to detect short-term variation and systematic change (from an established baseline) at a within-person level. However, the majority of available PROMs were designed to detect between-person differences, a snapshot of one point in time, rather than monitor change. This hurdle may help to explain why the literature is largely missing repeated measures studies of PRO to address the first step. To facilitate this type of research and avoid the necessarily time-consuming complexities of the development and validation of a new survey measure, the present study investigates whether the widely-used and disease-generic SF-36 can serve as a repeated PROM; that is, whether it can reliably detect person-level variation without sacrificing measurement properties as determined by its factor structure.

Our first research question asked whether the SF-36 is sensitive enough for the detection of short-term within-person variation. This was answered through inspection of ICC values for each item and subscale after seven consecutive days of responses. We found a wide range of ICC values, indicating that some items captured a greater proportion of daily dynamics relative to stable, between-person differences, than others. Visual inspection of scatterplots for a random sample further illustrated varying degrees of within-person variation across days and subscales. In particular, the Emotional Role Limitations, Mental Health, Social Functioning and General Health subscales revealed the largest magnitude of within-person variation, suggesting that these components of health may be key indicators for PROM monitoring, perhaps because they are more likely to be impacted by daily events and activities. The presence of day-to-day variation highlights the need to utilize repeated measurements in order to disaggregate within-person variations from between-person differences. Failing to account for these within-person fluctuations in health outcomes assumes that they are stable and prevents us from understanding the impact that daily variations in health have on the individual.

Our second research question asked whether the psychometric properties of the SF-36 were maintained during “off-label” use as a repeated PROM. That is, do items continue to load onto their respective subscales, and subscales onto summary components, to comprise the same latent factors yielded by standard use of the survey? To evaluate this, we compared the factor structure of the standard survey administered at baseline to that of the daily survey. We found no substantial differences between them, indicating that summarizing item responses by subscales and summary components is appropriate to monitor person-level change. However, the fit indices of both versions were sub-optimal. To evaluate the sources of model misfit, we inspected the modification indices and noted that the primary sources were in the Vitality and Mental Health subscales, particularly at the within-person level, such that the positive items loaded together. This is in line with the structure of most measures of positive and negative affect such as Watson’s Positive and Negative Affect Schedule [34, 39]. We evaluated an alternative factor structure for both the baseline and daily SF-36, allowing positive and negative Vitality and Mental Health items to load onto separate factors, but found that it did not substantially improve overall model fit (results not shown).

Our findings on the sub-optimal model fit of the SF-36 are consistent with previous work which has raised issue with the factor structure and construct validity obtained by the recommended orthogonal scoring procedure [25–29] and the reduction to summary component measures [27, 40–44]. Thus, although the daily SF-36 exhibited similar psychometric properties to the standard survey, sub-optimal fit indices in both cases lead us to recommend caution in using the SF-36 in its entirety as a repeated PROM. However, while the overall multifactor model of the SF-36 exhibited sub-optimal fit indices, many of the subscales demonstrated acceptable to good reliability estimates when examined independently. Researchers may find utility in focusing on improving and expanding the specific subscales for use in certain contexts. Including additional items for the subscales that contained only two items and reconsidering the arrangement of the Mental Health and Vitality subscales into positive and negative affect subscales (e.g., [34, 39]) are two potentially fruitful avenues to explore.

A limitation of this study is the relatively healthy sample, which may explain why some items on the daily SF-36 (e.g., *walk mile* under Physical Functioning, *magnitude* under Bodily Pain) exhibited little within-person variation. Alternatively, this may be because some health-related factors are simply unlikely to show short-term change, or because some items are not sensitive enough to detect the occurrence of short-term changes. A second limitation is the extent to which sample heterogeneity may have contributed to the overall poor fit. There is some evidence that the SF-36 factor structure may differ among patient subgroups, particularly those with comorbidity (e.g., [42, 44, 45]); that is, some survey subscales may have disease-specific relationships with either summary score. However, this has only been found to be a concern for the two-factor structure of the survey so is unlikely to have substantially affected our results given our focus on the eight-factor structure.

This study extends prior research on PRO assessment to consider the utility of the SF-36 as a PRO measure for repeated administration. This was accomplished through evaluation of its factor structure at the within-person level. We found that the SF-36 modified for repeated administration has a similar factor structure to the standard version, indicating maintenance of measurement properties when used “off-label,” though model fit remained sub-optimal. However, many subscale reliabilities ranged from acceptable to good at both the within-person and between-person levels. Therefore, while we conclude that the SF-36 in its entirety may not be an adequate measure for repeated PRO assessment, we recommend future work to examine the utility of the subscales in specific contexts, as well as the within-person factor structure of other PROMs currently in use (e.g., [20–22]). This is an important first step in the measurement of daily PRO assessments in primary health care. Future research can build upon this work in moving toward the goal of regular in-clinic patient assessment and early detection of the cognitive, behavioral and physical changes that characterize potentially reversible conditions and personalizing interventions and health care. This may be more easily facilitated by the adaptation and integration of existing measures than the development of new surveys.

## Conclusions

Many of the subscales of the modified SF-36 for repeated daily assessment were found to be sufficiently reliable for use in repeated measurement designs incorporating PROMs, though the overall scale may not be optimal. We encourage future work to investigate the utility of the subscales in specific contexts, as well as the measurement properties of other existing PROMs when administered in a repeated measures design. The development and integration of new measures for this purpose may ultimately be necessary.

## Abbreviations

CFI: comparative fit index
ICC: intra-class correlation
PRO: patient-reported outcome
RMSEA: root-mean-square-error of approximation
SF-36: RAND 36-Item Short Form Health Survey 1.0 *Statistics*
SRMR: standardized root-mean-square
